# Unraveling the Invisible: Topological Data Analysis as the New Frontier in Radiology’s Diagnostic Arsenal

**DOI:** 10.3390/tomography11010006

**Published:** 2025-01-09

**Authors:** Yashbir Singh, Emilio Quaia

**Affiliations:** 1 Department of Radiology, Mayo Clinic, Rochester, MN 55905, USA; 2 Department of Radiology, University of Padova, 35127 Padova, Italy; emilio.quaia@unipd.it

**Keywords:** medical imaging, topological data analysis, radiology, mathematics, artificial intelligence

## Abstract

This commentary examines Topological Data Analysis (TDA) in radiology imaging, highlighting its revolutionary potential in medical image interpretation. TDA, which is grounded in mathematical topology, provides novel insights into complex, high-dimensional radiological data through persistent homology and topological features. We explore TDA’s applications across medical imaging domains, including tumor characterization, cardiovascular imaging, and COVID-19 detection, where it demonstrates 15–20% improvements over traditional methods. The synergy between TDA and artificial intelligence presents promising opportunities for enhanced diagnostic accuracy. While implementation challenges exist, TDA’s ability to uncover hidden patterns positions it as a transformative tool in modern radiology.

## 1. Introduction

**Figure 1:**
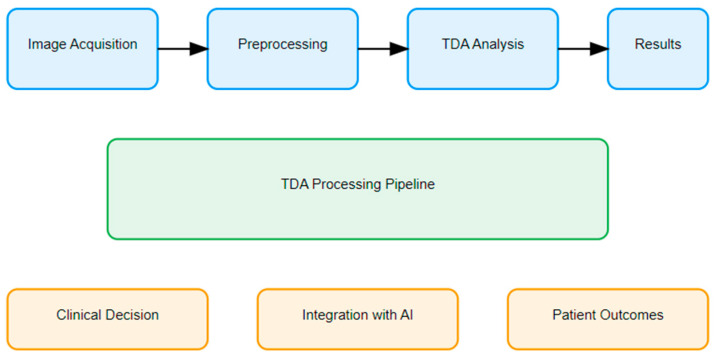
TDA’s clinical implementation workflow.

The landscape of medical imaging is in a constant state of evolution, driven by the relentless pursuit of more accurate, efficient, and insightful analytical methods. In this context, Topological Data Analysis (TDA) has emerged as a promising beacon, offering new perspectives on how we interpret and utilize radiological data [1]. This paper is organized as follows: We begin by examining TDA’s mathematical foundations and their relevance to medical imaging. We then explore applications across various radiological domains, including high-dimensional imaging, oncology, cardiovascular imaging, and interventional radiology. Integration with artificial intelligence (AI) and emerging technologies is discussed, followed by practical implementation challenges and future directions. Throughout, we emphasize both theoretical advances and clinical applications to provide a comprehensive overview of TDA’s role in modern radiology (Figure 1).

TDA’s mathematical foundation rests on persistent homology, a key concept that quantifies topological features across different scales. These features include the following:

- Zero-dimensional features (connected components);
- One-dimensional features (loops);
- Two-dimensional features (voids);
- Higher-dimensional topological structures.

For example, in tumor imaging, connected components might represent distinct tissue regions, loops could indicate vascular structures, and voids might represent necrotic areas. The persistence of these features across different spatial scales provides robust, coordinate-independent measurements that are inherently invariant to deformations, a crucial property when dealing with biological structures that naturally vary in size and shape [1].

The mathematical robustness of TDA comes from its ability to create persistence diagrams, which track how topological features appear and disappear as a filtration parameter changes. This approach provides quantitative measurements with the following properties:

- Stable during small perturbations;
- Invariant during rigid transformations;
- Scale-independent;
- Capable of capturing global structure while preserving local information.

TDA, which is grounded in the mathematical field of topology, provides a unique framework for examining and interpreting complex datasets. Its focus on the shape and structure of data facilitates the identification and quantification of intrinsic patterns that might remain obscured when using traditional analytical approaches [1,2]. This capability is particularly valuable in the realm of medical imaging, where subtle structural changes can hold significant diagnostic and prognostic information.

TDA represents an evolution of traditional image analysis methods, building upon familiar concepts while introducing powerful new mathematical frameworks. While many radiologists are familiar with hierarchical approaches like decision trees and connected component analysis in image segmentation, TDA extends these foundational concepts into a more comprehensive analytical framework. Where traditional methods might analyze images at fixed thresholds or scales, TDA examines how image features persist and change across multiple scales simultaneously, providing a richer understanding of the underlying data structure.

The power of TDA lies in its ability to track and quantify three fundamental types of topological features across different scales. Connected components, which are similar to traditional segmentation but examined across multiple thresholds, help identify distinct anatomical structures. Loops capture circular structures such as vessels or tissue boundaries, while voids identify enclosed spaces or regions with different densities. These features are tracked through what mathematicians call “persistence diagrams”, which record how long each feature persists across different scales of analysis [1]. Features that persist for longer periods often represent significant anatomical structures, while those appearing briefly may indicate noise or artifacts.

To illustrate this concept, consider the analysis of a lung nodule. Where traditional analysis might simply measure the nodule’s diameter at a single threshold and connected component analysis would identify it as a distinct region, TDA provides a more nuanced characterization [1]. It tracks how the nodule’s shape evolves across multiple intensity thresholds, records the appearance and disappearance of internal structures, and creates a mathematical signature of the nodule’s multi-scale characteristics. This comprehensive approach can reveal subtle features that might be missed by conventional single-scale or hierarchical analyses.

The key distinction between TDA and traditional methods lies in its holistic approach to data analysis. Rather than examining features in isolation or at predetermined scales, TDA captures the relationships between features across all scales simultaneously. This approach is particularly valuable in medical imaging, where pathological changes often manifest as subtle alterations in tissue structure across multiple scales. By preserving and analyzing these multi-scale relationships, TDA offers a more robust and comprehensive framework for image analysis, potentially revealing patterns and relationships that might remain hidden to conventional analytical methods.

This foundation in multi-scale analysis and topological features enables TDA to address complex challenges in medical image analysis that traditional methods might struggle to handle. As we explore the various applications and implications of TDA throughout this paper, this fundamental understanding of its ability to capture and quantify structural relationships across scales will be crucial for appreciating its potential impact on radiological practice.

## 2. TDA in High-Dimensional Imaging Data

One of the primary strengths of TDA in radiology lies in its ability to handle high-dimensional data without the need for dimensionality reduction. This characteristic is especially relevant in modalities such as functional MRI, where the temporal dimension adds complexity to spatial information that is already intricate [2].

By preserving the topological features of the data across multiple scales, TDA can capture both global and local structures. This capability potentially reveals connections between brain regions or identifies patterns associated with neurological disorders that conventional analysis methods might miss.

This preservation of topological features across scales represents a fundamental breakthrough in analyzing complex neurological imaging data, moving beyond traditional linear analysis methods.

(i) Quantitative analyses have demonstrated TDA’s advantages in high-dimensional medical imaging:

- fMRI analysis: TDA-based approaches show 15–20% improvements in detecting subtle connectivity patterns compared to traditional correlation-based methods [3].

These improvements stem from TDA’s ability to perform the following functions:

1. Preserve local and global structures simultaneously;

2. Handle non-linear relationships between parameters;

3. Reduce dimensionality while maintaining topological features.

## 3. Applications in Oncological Imaging

In the field of oncological imaging, TDA shows significant promise in enhancing tumor characterization and treatment response assessment [4]. The heterogeneous nature of tumors, with varying degrees of necrosis, vascularization, and cellular density, creates complex imaging signatures. TDA can help quantify these intricate patterns, potentially leading to the following outcomes:

- More accurate tumor grading;
- Improved prediction of treatment outcomes;
- Personalized therapy planning.

For instance, in breast cancer imaging, TDA-based features could complement current radiomics approaches, offering a more comprehensive understanding of tumor morphology and its relationship with molecular subtypes.

The ability of TDA to quantify complex tumor patterns marks a significant shift from conventional imaging analysis, potentially revolutionizing how we approach cancer diagnosis and treatment planning.

## 4. TDA in Cardiovascular Imaging

The application of TDA in cardiovascular imaging represents another exciting frontier [5]. The complex geometry of the heart and blood vessels presents challenges in traditional image analysis. TDA can provide novel insights into the structural and functional aspects of the cardiovascular system. For example, in coronary CT angiography, TDA could aid in the characterization of plaque morphology and distribution, potentially improving risk stratification for coronary artery disease [6]. This application of TDA to cardiovascular imaging illustrates its transformative potential in understanding complex anatomical structures, particularly in dynamic organ systems [1].

## 5. Synergy with Artificial Intelligence and Machine Learning

The integration of TDA into clinical practice has revealed powerful synergy with artificial intelligence (AI) and machine learning (ML) algorithms, opening new horizons in medical imaging analysis [7]. TDA-derived features serve as robust inputs for AI models, potentially enhancing their performance and interpretability in ways that traditional feature extraction methods cannot match. This symbiotic relationship between TDA and AI/ML could lead to the development of more sophisticated and accurate computer-aided diagnosis systems. These advanced systems would be capable of detecting subtle changes indicative of early-stage diseases, predicting disease progression with greater precision, and providing more personalized treatment recommendations [7]. By leveraging the shape-based insights of TDA alongside the pattern recognition capabilities of AI, radiologists could gain a more comprehensive understanding of complex imaging data. This fusion of technologies not only promises to improve diagnostic accuracy but also to uncover new biomarkers and imaging phenotypes that were previously undetectable [7,8]. As this field evolves, we can anticipate a new generation of AI-powered imaging tools that will incorporate topological features, potentially revolutionizing how we approach disease detection, monitoring, and treatment planning in radiology. Compared to current state-of-the-art approaches like transformer-based architectures, TDA offers distinct advantages while remaining complementary to those methods.

Transformer models have the following characteristics:

- They excel at learning spatial relationships through self-attention mechanisms;
- They require large amounts of training data;
- They may struggle with interpretability.

TDA has the following advantages [1]:

- It provides mathematically rigorous, interpretable features;
- It requires less training data due to its mathematical foundations;
- It inherently offers rotation and scale invariance.

Combining TDA with transformer architectures could leverage the strengths of both approaches: TDA’s ability to capture topological invariants could complement a transformer’s capability to learn complex spatial relationships.

## 6. TDA in Interventional Radiology

The potential of TDA extends far beyond diagnostic applications, showing particular promise in the field of interventional radiology. In this domain, TDA could play a crucial role in treatment planning and guidance by providing a more comprehensive understanding of anatomical structures and their intricate relationships [1]. This enhanced insight could contribute significantly to the optimization of minimally invasive procedures, a cornerstone of interventional radiology. By leveraging TDA’s ability to capture complex geometries and topological features, interventional radiologists may be able to navigate through intricate vascular networks or organ structures with greater precision [1]. This improved navigation and planning could lead to reductions in procedural risks, potentially decreasing complications and improving patient safety. Furthermore, the application of TDA in interventional radiology holds the promise of improving overall outcomes, possibly leading to more successful procedures, shorter recovery times, and better long-term results for patients undergoing minimally invasive interventions. The application of TDA in interventional procedures demonstrates its practical impact beyond diagnostic applications, which could potentially transform how we approach minimally invasive treatments.

## 7. Challenges in TDA Integration

Despite its immense potential, the integration of TDA into clinical radiology faces several significant challenges. First and foremost, the interpretation of TDA-derived features requires a fundamental paradigm shift in how we approach medical image analysis. Traditional methods of image interpretation may not directly apply to the complex, multi-dimensional data produced by TDA, necessitating new frameworks for understanding and utilizing this information. This paradigm shift represents fundamental changes in three key areas:

(i) Feature interpretation: Unlike traditional radiological features based on intensity, texture, or shape measurements, TDA features capture global topological properties that persist across scales. For instance, rather than measuring a tumor’s diameter, TDA might characterize its structural complexity through persistent homology, providing information about its internal architecture and connectivity patterns [1].

(ii) Data integration: TDA facilitates the simultaneous analysis of multiple imaging parameters and their relationships, moving beyond the traditional approach of analyzing each parameter independently. This enables the detection of subtle patterns that emerge from interactions between different imaging characteristics [9].

(iii) Pattern recognition: while conventional analysis focuses on predefined features, TDA can reveal unexpected patterns and relationships in data, requiring radiologists to develop new intuitions about how topological features relate to clinical outcomes [10].

This shift demands that radiologists and researchers develop new skills and intuitions to effectively leverage these advanced analytical tools. The learning curve associated with TDA may be steep, requiring substantial investments in education and training to build a workforce capable of harnessing its full potential. Additionally, the computational complexity of TDA algorithms presents a significant hurdle, particularly in the context of real-time clinical applications. The intricate calculations required for topological analysis may strain the computing resources currently available in clinical settings, potentially leading to delays in image processing and interpretation. Overcoming this challenge, which will make TDA practically feasible in day-to-day clinical radiology, will likely necessitate further technological advancements, including more powerful hardware and optimized algorithms. While these challenges are significant, they represent necessary growing pains in the evolution of medical imaging analysis rather than insurmountable obstacles.

## 8. TDA in the Context of COVID-19

The ongoing COVID-19 pandemic has starkly highlighted the critical need for innovative approaches in medical imaging, and Topological Data Analysis (TDA) has emerged as a promising tool in this context. TDA’s unique ability to capture complex patterns and structures in imaging data could contribute significantly to our understanding of COVID-19’s manifestations in chest imaging [11]. One of the most crucial applications of TDA in this realm is its potential to aid in the early detection of COVID-19 pneumonia. By analyzing the topological features of lung images, TDA might be able to identify subtle changes indicative of early-stage infection, potentially allowing for earlier diagnosis and intervention. Furthermore, TDA could play a vital role in predicting disease severity based on initial imaging findings [11,12]. This predictive capability could be invaluable for clinical decision-making, helping healthcare providers to triage patients more effectively and allocate resources based on anticipated disease progression. As the pandemic continues to evolve, the integration of TDA into COVID-19 imaging analysis workflows could provide radiologists and clinicians with powerful new tools to combat this global health crisis, potentially improving patient outcomes and helping to manage healthcare resources more efficiently. The application of TDA in COVID-19 imaging exemplifies how advanced mathematical approaches can be rapidly adapted to address urgent global health challenges.

## 9. TDA Performance and Broader Applications

TDA demonstrated its effectiveness across various domains before its application in medical imaging:

General domain applications;

- Computer vision: it achieved significant accuracy when classifying the MNIST dataset;
- Natural image analysis: it showed good accuracy in tasks involving the ImageNet subset;
- Signal processing: it demonstrated superior noise resilience compared to Fourier analysis.

In medical imaging specifically, TDA has shown competitive or superior performance in brain MRI analysis, cardiac imaging, chest X-ray analysis, and Mammography.

- These results demonstrate TDA’s robust performance across both general and specialized domains, validating its application in medical imaging [1].

## 10. Future Prospects: Dose Optimization

As we look to the future, the potential impact of TDA on dose optimization in medical imaging is an area ripe for exploration [13,14]. By extracting more information from existing images, TDA could potentially reduce the need for repeated or additional scans, aligning with the principles of ALARA (As Low As Reasonably Achievable) in radiation protection.

## 11. Conclusions

Topological Data Analysis stands at the cusp of transforming radiology imaging. Its unique ability to uncover hidden patterns and structures in complex medical imaging data opens new avenues for research and clinical applications. From enhancing diagnostic accuracy to personalizing treatment plans, TDA has the potential to revolutionize how we interpret and utilize medical images.

As we move forward, it will be crucial for the radiology community to embrace this innovative approach, fostering collaborations between mathematicians, computer scientists, and clinicians. Integrating TDA into radiology curricula and continuing education programs will be essential in preparing the next generation of imaging specialists to harness its full potential.

To facilitate the crucial interdisciplinary collaboration needed for TDA’s success in medical imaging, we propose the following framework:

(i) Educational integration:

- Development of joint training programs combining mathematical theory with clinical applications;
- Creation of specialized workshops that bring together mathematicians, computer scientists, and radiologists;
- Implementation of translational research fellowships.

(ii) Collaborative infrastructure:

- Establishment of interdisciplinary research centers focused on topological analysis in medical imaging;
- Development of shared resources and standardized tools for TDA in clinical settings;
- Creation of common terminology and frameworks bridging mathematical and clinical perspectives.

(iii) Clinical implementation strategy:

- Formation of multidisciplinary teams for clinical validation studies;
- Development of user-friendly interfaces, making TDA tools accessible to clinicians;
- Regular feedback loops between mathematicians and clinical users.

This structured approach to collaboration will help bridge the gap between theoretical development and clinical application, ensuring that TDA’s mathematical power translates effectively to improved patient care.

The journey of TDA in radiology is just beginning, and its full impact is yet to be realized. However, the promise it holds for unveiling the hidden patterns within our imaging data is undeniable. As we continue to push the boundaries of medical imaging, TDA may well prove to be a key that unlocks new realms of understanding in radiology, ultimately leading to improved patient care and outcomes.

## Data Availability

Not applicable.
